# Evaluating the accuracy of HIV-1 viral load testing using near point of care Xpert HIV-1 system at Solwezi general hospital, Zambia

**DOI:** 10.1186/s13104-025-07387-w

**Published:** 2025-07-21

**Authors:** Keembe Siakantu, David Chisompola, Aaron Tembo Konzani

**Affiliations:** 1 Pathology Laboratory Dept, Solwezi General Hospital, Solwezi, Zambia; 2 Pathology Laboratory Dept, Arthur Davison Children’s Hospital, Ndola, Zambia; 3 Pathology Laboratory Dept, Tropical Gastroenterology and Nutrition group, Lusaka, Zambia

**Keywords:** Accuracy, Diagnosis, GeneXpert, HIV-1, Hologic panther, Solwezi, Zambia

## Abstract

**Objective:**

HIV-1 Viral Load Testing plays a crucial role in the management of HIV/AIDS patients by quantifying the presence of HIV-1 RNA in the blood, which directly relates to viral replication and disease progression. Monitoring viral load enables healthcare professionals to evaluate the effectiveness of Anti-Retroviral Therapy, adapt treatment strategies, and assess the overall health status of individuals with HIV. This was a cross-sectional study to evaluate analytical performance (accuracy, precision and linearity) of the near point of care Xpert HIV-1 viral load.

**Results:**

The analytical performance of the GeneXpert assay to the conventional Hologic Panther system was strong. Specifically, the GeneXpert demonstrated a sensitivity, specificity, and accuracy of 87.6%, 100%, and 98.5%, respectively, when compared to the Hologic Panther. A strong correlation between the two assays was evident (*r* = 0.97, *p* < 0.0001). Additionally, precision, as indicated by the coefficient of variation (CV), for high viral load was 1.38% for GeneXpert while for low viral load, it was 2.59%. In the linearity analysis, all data points remained within acceptable ranges, yielding correlation coefficients ≥ 0.99 for both assays.

**Conclusion:**

The Xpert HIV-1 viral load assay demonstrates high efficacy in accurately identifying and quantifying HIV-1 viral loads, making it a valuable tool for HIV diagnosis and monitoring. This reliability makes it particularly suitable for critical populations requiring timely and accurate viral load monitoring.

## Introduction

### Background

The Human Immunodeficiency Viruses (HIV) has a significant global impact, and it has been estimated that 38 million people worldwide are living with HIV/AIDS in 2020 [1]. In Africa, particularly in Zambia, the burden of HIV remains alarmingly high. As of 2020, approximately 1.2 million people in the country were receiving treatment for HIV, highlighting the ongoing public health challenge and the critical need for sustained intervention efforts [2].

Zambia has been significantly impacted by the HIV-1 epidemic, with approximately 11.0% of the population aged 15 and older living with HIV in 2021. In Northwestern Province, 6.8% of the province’s population is affected by the virus [3]. This indicates a considerable number of individuals in Solwezi who require effective management and monitoring of the HIV infection.

Viral load (VL) testing using the near point-of-care (POC) GeneXpert platform (Cepheid, Sunnyvale, CA, USA) with the Xpert HIV-1 quantitative assay offers the advantage of being used closer to patients. It is easy to use, gives quick results, is cost-effective, and has the potential to serve as an alternative to centralized viral load testing platforms [4]. POC has been introduced for rural populations to expand access to clinical laboratory testing by eliminating the need for sample transportation, laboratory and data management infrastructure, and highly trained staff [5]. Therefore, ensuring access to reliable and accurate viral load testing is vital in improving patient outcomes and reducing HIV transmission rates in Solwezi and similar high-burden areas [4].

The GeneXpert system was recently adopted to reduce the turnaround time for the urgent population such as pregnant and breastfeeding women and children from 0 to 14 years in point of care facilities [6]. Thereby, adding the Xpert HIV-1 VL to the list of analysers employed for HIV-1 viral load testing in Zambia [6]. However, the evaluation of Xpert HIV-1 VL systems’ performance and accuracy remains unclear. Differences in detection methodology, sample handling, operator skill level, and other factors may result in discrepancies between the conventional systems’ and point of care measurements. Such discrepancies might not only affect individual patient care but could also have broader implications for public health, especially in settings where the HIV prevalence is high [7]. Therefore, it is critical to understand and address this knowledge gap to optimize HIV-1 viral load testing and thus improve patient outcomes and public health strategies. This study aimed to evaluate analytical performance and accuracy of HIV-1 viral load measurements of the GeneXpert system in comparison to the Hologic Panther system.

## Methods

### Study design and setting

We conducted a cross-sectional study at Solwezi General Hospital Laboratory in Solwezi District, the main referral centre for HIV Viral load monitoring in the North-western Province of Zambia.

### Sample size

A total of 159 EDTA plasma remnant samples collected from individuals living with HIV who were receiving antiretroviral therapy (ART) and undergoing routine viral load monitoring at Solwezi General Hospital using the Hologic Panther [8], were selected. A total of 117 samples were used to assess sensitivity and specificity, while 32 samples were analysed for the correlation coefficient. Additionally, precision testing was conducted using 5 low HIV viral load samples and 5 high HIV viral load samples, each processed in 5 replicates over 5 consecutive days. Low viral load was defined as ≤ 999 copies/mL, while high viral load was defined as ≥ 100,000 copies/mL. For linearity assessment, five pooled samples were diluted and processed to evaluate the Pearson correlation coefficient. All results were converted to log10 copies/mL for statistical analysis.

### Sample collection and storage

Plasma was extracted from whole-blood samples collected in EDTA-containing tubes and separated by centrifugation at 1,500 × g for 10 min. An initial 0.6 mL aliquot was tested using the Aptima HIV-1 assay as part of routine HIV-1 viral load monitoring. Additional plasma aliquots were stored at − 80 °C in volumes designated for the Xpert HIV-1 assay, undergoing a single freeze-thaw cycle before analysis. On the day of testing, the aliquots were thawed, vortex-mixed, and analysed for viral load quantification.

### Test method

The Xpert HIV-1 Viral Load is a cartridge-based, total nucleic acid real-time polymerase chain reaction (RT-PCR) test for the detection and quantification of Human Immunodeficiency Virus type 1 (HIV-1) RNA in human plasma from HIV-1 infected individuals, using the automated GeneXpert Instrument Systems (Cepheid, Sunnyvale, CA, USA) [9]. It is a near point of care test that can quantify HIV-1 RNA over the range of 40 to 10,000,000 copies/mL. The Xpert HIV-1 VL assay targets the Long Terminal Repeats (LTR) gene of the HIV-1 RNA [10]. A volume of 1000 µl of plasma specimen is needed to perform the assay (if using the transfer pipette included in the kit, a minimum of 1.2 mL of plasma is required) [9]. This assay necessitates additional laboratory equipment for specimen preparation, such as a centrifuge and a refrigerator (if specimen storage is required), but it can still be conducted in laboratories with minimal resources. The equipment relies on a consistent power supply [9].

The Hologic Aptima HIV-1 Quant Dx viral load assay was conducted following the manufacturer’s guidelines and analysed using the fully automated Panther instrument system. The Aptima assays utilize real-time transcription-mediated amplification (RT-TMA) for the detection and quantification of HIV-1 RNA [11]. The Aptima HIV-1 Quant Dx assay targets the polymerase (pol) and LTR regions, has a minimal required sample volume of 0.7 mL of specimen, and reports quantitative HIV-1 results in a range of 30 to 10,000.000 copies/mL [11].

### Statistical analysis

Data collection was carried out using an Excel spreadsheet, which was later exported to IBM SPSS Statistics 22 and R Studio for statistical analysis. The precision of the Xpert HIV-1 VL platform was assessed using the Coefficient of Variation (CV) calculated through the one-way ANOVA method. The accuracy of the Xpert HIV-1 VL platform was determined using the Pearson correlation coefficient (R^2^), while its linearity was evaluated through the correlation coefficient (r).

The predefined thresholds for evaluating the acceptability of results were set as follows: Sensitivity ≥ 94.1%, Specificity ≥ 98.5%, Correlation coefficient (R-value) ≥ 0.941, precision with a coefficient of variation (CV) ≤ 3%, and Linearity with an R² ≥ 0.99 across serial dilutions [9].

### Ethical clearance

Ethical approval for the study was granted by the Mulungushi University School of Medicine Ethics Committee, and authorization to conduct the research was obtained from the management of Solwezi General Hospital.

## Results

### Sensitivity and specificity

A total number of 159 samples that were initially processed using the Aptima-HIV-1 assays were compared to the Xpert-HIV-1 VL samples. By using the quantification thresholds of the two assays, the VL results were classified as “Target not detected” or “Quantified”. The Xpert HIV-1 sensitivity and specificity using Aptima HIV-1 as reference method was 87.62% [95% CI, 79.01 – 93.17%], and 100.00% [95% CI, 79.95 – 100.00%] respectively, as shown below in Table 1.

**Table 1:** Sensitivity and specificity

	Estimated value (%) (*n* = 117)	95% Confidence interval	
		Lower limit	Upper limit
Sensitivity	87.62	79.01	93.17
Specificity	100	79.95	100

### Correlation between Xpert-HIV-1 VL and hologic panther

**Fig. 1:**
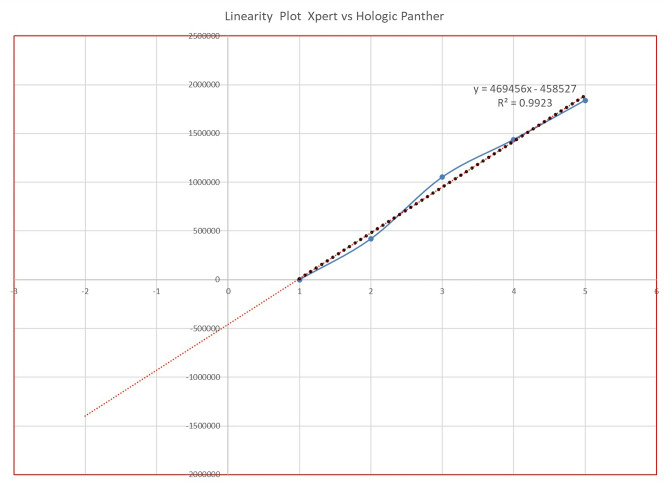
Xpert-HIV-1 VL and Hologic Panther correlation chart as shown here

The mean VL obtained by the Xpert-HIV-1 VL method was 2.96 log10 cp/mL with a Standard Deviation (SD) of 1.65 log10 cp/mL, while the mean VL obtained by the Hologic Panther was 3.06 log10 cp/mL (SD: 1.60 log10 cp/mL). A correlation between the two assays was observed (*r* = 0.97, *p* < 0.0001) as shown in Fig. 1.

### Precision results

The repeatability and within-laboratory mean results for samples with high and low concentrations demonstrated coefficient of variation (CV) values were 1.38 and 2.59, as highlighted in Tables 2 and 3.

**Table 2:** Precision for high positive viral load

Analyte	Test Type	Precision High Positive			Acceptability
		Mean	CV (%) Claim	Study CV (%)	
HIV Viral Load High Positive	Repeatability	5.18	3.0	0.71	Acceptable
	Within Lab precision		3.0	1.38	Acceptable

**Table 3:** Precision for low positive viral load

Analyte	Test Type	Precision Low Positive			Acceptability
		Mean	CV (%) Claim	Study CV (%)	
HIV Viral Load Low Positive	Repeatability	2.81	3.0	1.85	Acceptable
	Within Lab precision		3.0	2.59	Acceptable

### Linearity results

**Fig. 2:**
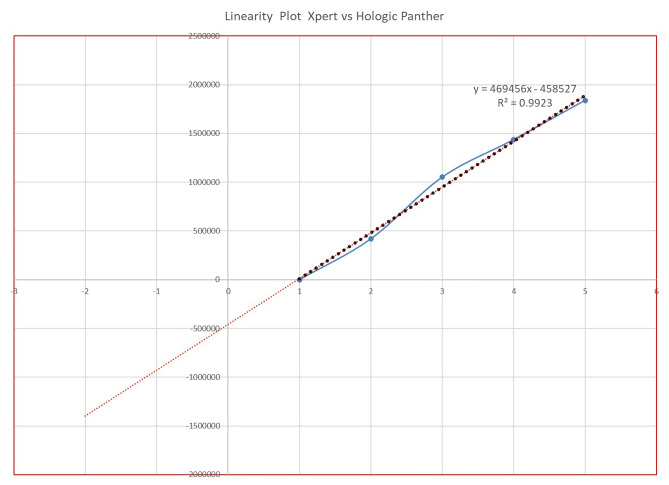
Xpert HIV-1 VL Linearity

All data points fell within the acceptable range, as demonstrated in Fig. 2.

## Discussion

The Xpert HIV-1 VL assay demonstrated an excellent analytical performance when compared to the conventional Hologic Panther system. Our evaluation confirms that the Xpert HIV-1 VL assay satisfies the performance criteria essential for clinical diagnosis. The Xpert HIV-1 VL assay achieved a sensitivity of 87.6% and a specificity of 100%, highlighting its reliability in distinguishing true positives and negatives. A strong correlation between the two assays was observed (*r* = 0.97, *p* < 0.0001). Reproducibility, assessed through the coefficient of variation (CV), was excellent, with a CV of 1.38% for high viral loads and 2.59% for low viral loads. Linearity analysis further confirmed the assay’s accuracy, with all data points falling within acceptable ranges and correlation coefficients (R²) consistently ≥ 0.992.

To our knowledge, this study is the first to evaluate the analytical performance of the Xpert HIV-1 assay compared to the Aptima HIV-1 assay. To support this analysis, we referenced the World Health Organisation (WHO) prequalification report and previous studies that assessed the Xpert HIV-1 assay against other analysers, such as the Roche Cobas assays.

Our study demonstrated a lower sensitivity compared to the findings of Kone et al. (2020) and Wesolowski et al. (2020), who reported sensitivities of 93.1% and 97.9%, respectively, when evaluating the Xpert HIV-1 VL assay against the Roche Cobas system [7, 10]. This discrepancy may be attributed to differences in sample handling. Our study utilized stored remnant plasma, which could have undergone RNA degradation, potentially affecting detection rates. Despite this, our study achieved a better specificity than what was reported by Kone et al. (2020) and by the WHO prequalification report, highlighting the validity of our assay in correctly identifying true negatives [7, 9].

Our findings on precision outperformed those reported by Woo et al. (2024), as our CV for precision was consistently below 5% [12]. This is a notable improvement over Smita et al. (2017), who reported CVs of 4.15% and 3.52% [13]. Furthermore, Gous et al. (2016), in their comparison of the Xpert HIV-1 VL assay with the Roche Cobas TaqMan v2 and Abbott HIV-1 assays, reported CVs of 1.5% and 0.9% [14]. Our findings aligned with these values for high-positive samples, although our CV for low-positive samples was higher than Gous et al., indicating potential areas for further optimization. Our CV for the low positive was still lower than what the WHO Prequalification of In Vitro Diagnostics report for 2023 for the evaluation of Xpert HIV-1 VL [9].

Our study also demonstrated a stronger correlation (*r* > 0.97) between the evaluated assays than the findings of Smita et al. (2017), who reported a correlation coefficient of 0.886 when comparing the GeneXpert assay with the Abbott assay [13]. Similarly, our findings showed superior performance when comparing the Xpert HIV-1 assay to the Hologic Panther system, outperforming the correlation reported by Mor et al. (2015) (*r* > 0.89) [15].

The linearity of the Xpert HIV-1 VL assay in our study was consistent with Wesolowski et al. (2020) and by the WHO prequalification, who reported a correlation coefficient (R²) > 0.99 when comparing the assay with the Roche Cobas Ampliprep/Cobas TaqMan system [9, 10].

The differences in detection thresholds between the Xpert HIV-1 VL (40 copies/mL) and Hologic Panther (30 copies/mL) assays may partially account for the observed sensitivity of 87.6%. Samples with viral loads near the lower limit of detection (30–40 copies/mL) could be quantified by the Panther but classified as “target not detected” by the Xpert HIV-1, leading to a reduced apparent sensitivity of the latter. This aligns with prior studies reporting sensitivity variations when comparing assays with various thresholds [9, 10]. Clinically, this difference is unlikely to compromise patient management, as both thresholds are well below the WHO-defined virologic failure threshold (≥ 1,000 copies/mL). However, in research settings requiring ultra-sensitive detection (e.g., HIV cure studies), the Panther’s lower threshold may offer an advantage. Future evaluations should include stratification by viral load ranges to clarify the Xpert’s performance near its detection limit.

The Xpert HIV-1 VL assay offers exceptional accuracy and a significantly reduced turnaround time, making it an ideal choice for clinical laboratories serving critical populations. Moreover, its single-cartridge design minimizes additional costs and manpower typically required from conducting VL testing and reduces the need for extensive infrastructure required by conventional molecular platforms.

## Conclusion

The GeneXpert HIV-1 viral load assay demonstrates high efficacy in accurately identifying and quantifying HIV-1 viral loads, making it a valuable tool for HIV diagnosis and monitoring. Its strong analytical performance, combined with its compatibility with conventional equipment systems, positions it as a flexible and dependable solution for resource-limited settings such as Zambia. This reliability makes it particularly suitable for critical populations requiring timely and accurate viral load monitoring. Future research should focus on optimizing assay performance and addressing any limitations to further enhance its utility in diverse healthcare settings.

## Limitations

This study had several limitations. First, the use of stored specimen remnants may have introduced the potential for specimen degradation, which could have contributed to the lower sensitivity observed. Second, the study was limited to a single comparator assay, the Hologic Panther VL system, which, while robust, may be less accurate than other widely used platforms such as the Roche Cobas TaqMan HIV-1 assay. Lastly, the limited availability of studies comparing the performance of the Xpert HIV-1 VL assay to the Hologic Panther system restricts the broader contextualization of our findings. Future research incorporating fresh specimens and multiple comparator assays is recommended to validate and expand upon these results.

## Data Availability

The datasets used and/or analysed during the current study are available from the corresponding author on reasonable request.
